# Novel Prognostic Markers in Triple-Negative Breast Cancer Discovered by MALDI-Mass Spectrometry Imaging

**DOI:** 10.3389/fonc.2019.00379

**Published:** 2019-05-14

**Authors:** Leo Phillips, Anthony J. Gill, Robert C. Baxter

**Affiliations:** ^1^Hormones and Cancer Group, University of Sydney, Kolling Institute, Royal North Shore Hospital, St Leonards, NSW, Australia; ^2^Cancer Diagnosis and Pathology Group, University of Sydney, Kolling Institute, Royal North Shore Hospital, St Leonards, NSW, Australia

**Keywords:** triple-negative breast cancer, MALDI MS, tissue imaging, tryptic peptide, prognosis

## Abstract

There are no widely-accepted prognostic markers currently available to predict outcomes in patients with triple-negative breast cancer (TNBC), and no targeted therapies with confirmed benefit. We have used MALDI mass spectrometry imaging (MSI) of tryptic peptides to compare regions of cancer and benign tissue in 10 formalin-fixed, paraffin-embedded sections of TNBC tumors. Proteins were identified by reference to a peptide library constructed by LC-MALDI-MS/MS analyses of the same tissues. The prognostic significance of proteins that distinguished between cancer and benign regions was estimated by Kaplan-Meier analysis of their gene expression from public databases. Among peptides that distinguished between cancer and benign tissue in at least 3 tissues with a ROC area under the curve >0.7, 14 represented proteins identified from the reference library, including proteins not previously associated with breast cancer. Initial network analysis using the STRING database showed no obvious functional relationships except among collagen subunits COL1A1, COL1A2, and COL63A, but manual curation, including the addition of EGFR to the analysis, revealed a unique network connecting 10 of the 14 proteins. Kaplan-Meier survival analysis to examine the relationship between tumor expression of genes encoding the 14 proteins, and recurrence-free survival (RFS) in patients with basal-like TNBC showed that, compared to low expression, high expression of nine of the genes was associated with significantly worse RFS, most with hazard ratios >2. In contrast, in estrogen receptor-positive tumors, high expression of these genes showed only low, or no, association with worse RFS. These proteins are proposed as putative markers of RFS in TNBC, and some may also be considered as possible targets for future therapies.

## Introduction

Triple-negative breast cancer (TNBC) is defined by low or absent expression of receptors for estrogen (ER) and progesterone (PR), without overexpression of the human epidermal growth factor (EGF) receptor-2 (HER2) (1). Owing to a lack of well-characterized treatment targets, women with TNBC tumors have fewer treatment options than are available for other breast cancer types, and are typically treated with chemotherapy. Relapse is common, usually in the first 5 years, leading to relatively poor survival outcomes. There are currently no well-established prognostic markers used in TNBC, and the development of new prognostic indicators, to complement basal markers such as cytokeratins (CK) 17 (2) and 5/6 (3), might be of benefit to the clinical management of the disease (4–6).

In this study we have used MALDI-TOF mass spectrometry imaging (MALDI MSI) of peptides, generated from endogenous proteins by tryptic digestion, to discover proteins with differential relative abundance in TNBC tissue compared to adjacent non-cancer tissue, with the aim of developing new markers with potential to be used in disease prognosis. MALDI MSI offers a wealth of information for analyzing the spatial distribution of biological molecules and the state of chemical modification present in proteins or peptides within tissue sections (7–9). While MALDI MSI was originally developed for analysis of intact proteins, peptides, and other small molecules present in frozen sections (10), the ability to analyze the distribution of peptides generated by tryptic digestion of proteins in formalin-fixed, paraffin-embedded (FFPE) archival tissues has greatly expanded both the clinical sample availability and the protein mass range accessible to analysis by this technique (11, 12).

## Materials and Methods

### Materials

Acetonitrile (ACN) and other solvents were obtained from commercial sources at the highest purity available and used without further purification. Proteomics grade Trypsin Gold (V5280) was purchased from Promega (Melbourne, VIC). CHCA (α-cyano-4-hydroxycinnamic acid), peptide standard mix, indium tin oxide (ITO) treated glass slides, and AnchorChip target plates (384 and 1,536 samples) were obtained from Bruker Daltonics (Preston, VIC, Australia). RapiGest SF surfactant was from Waters (Rydalmere, NSW, Australia). ZipTip C18 micropipet tips were from Merck-Millipore (Bayswater, VIC). The Pierce BCA (bicinchoninic acid) protein assay kit was from Thermo Fisher (Scoresby, VIC, Australia). Phenex-RC membrane syringe filters (4 mm, 0.2 μm) were from Phenomenex (Lane Cove, NSW, Australia).

### Patient Specimens

This study has been approved by the Human Research Ethics Committee of the Northern Sydney Local Health District (NSLHD), NSW, Australia. Patient consent was not required for the analysis of deidentified archival FFPE samples. Ten FFPE blocks containing TNBC tissue from 10 patients were obtained from the Department of Surgical Pathology, University of Sydney, NSW, Australia. The samples were deidentified with no clinical information provided apart from their triple-negative status. Sections of 10 μm were cut with a microtome and mounted onto ITO treated glass slides. Adjacent sections were mounted onto conventional glass slides for hematoxylin and eosin staining to permit histopathology evaluation of the tumor location.

### Slide Preparation for MSI Analysis

Mounted FFPE sections were deparaffinized in two changes of xylene (15 min each) and hydrated with a graded series of ethanol dips (2 min each: 100, 100, 95, 70, 0, 0%). Antigen retrieval was performed with 10 mM citrate acid buffer pH 6.0 using a Dako Cytomation Pascal pressure cooker (Model S2800, Dako, NSW, Australia), at 121°C for 16 min followed by 90°C for 16 min. Sections were then cooled to room temperature and washed with 10 mM ammonium bicarbonate (ABC) for 1 min. The ITO mounted tissue was digested using the ImagePrep automatic vibrational nebulizer (Bruker Daltonics) spraying a 0.1 μg/μL solution of trypsin in 20 mM ABC. Spraying was performed at room temperature using the ImagePrep's default “Trypsin Deposition” method. ITO slides were then incubated 2 h at 40°C in a humidified container. Internal peptide calibrants for mass calibration (13)—angiotensin II, angiotensin I, substance P, bombesin, ACTH(1–17), ACTH(18–39), somatostatin 28 (Bruker Daltonics)—were sprayed onto the slides and matrix solution of 7 mg/mL CHCA in 50% ACN/0.2% trifluoroacetic acid (TFA) was then sprayed using standard CHCA settings.

### Peptide Extraction for LC-MALDI Analysis

To extract tissues for LC-MALDI peptide analysis, slides were subjected to antigen retrieval and vacuum dried for 1 h, then the tissue was removed from the slide, placed in a 1.5 mL tube in 500 μl of 0.2% RapiGest SF surfactant in 50 mM ABC, vortexed, sonicated 10 min, then tris(2-carboxyethyl)phosphine was added to a final concentration of 5 mM. Samples were heated for 30 min at 60°C, cooled to room temperature, and alkylated with 15 mM iodoacetamide for 30 min in the dark. BCA assays were performed to determine protein concentrations. Trypsin was added at 1 μg per 50 μg protein, and incubated overnight at 37°C. TFA was added to the digested protein samples to a concentration of 0.5% (pH < 2) and incubated at 37°C for 40 min. Samples were centrifuged at 13,200 rpm for 10 min, and supernatants were collected and freeze-dried. Samples were reconstituted with 2% ACN/0.1% TFA, centrifuged at 13,200 rpm (16,110 × g; 45-24-11 rotor, Eppendorf, North Ryde, NSW, Australia), and supernatants were passed through a 0.2 μm filter into HPLC vials.

### MALDI MS Imaging Data

Each of the 10 H&E stained sections was examined by a pathologist (AJG) and regions of each section were designated cancerous, benign or common (both cancerous and benign tissue present). Adjacent sections on ITO slides were used to acquire imaging mass spectra from each region. Spectra were acquired using a Bruker UltrafleXtreme TOF-TOF MALDI mass spectrometer equipped with a SmartBeam 2000 Hz laser (Bruker Daltonics) in positive ion reflectron mode. Mass spectra were collected in the *m/z* range 700–3,500, with a spatial resolution of 50 μm. FlexImaging 4.1 (Build 116) was used to drive flexControl 3.4 (Build 125) during the acquisition. Data were visualized using flexImaging software (Bruker Daltonics). Spectral processing was performed using flexAnalysis 3.4 (Build 76) and SCiLS Lab 2014b (version 2.02.5378) software. MSI data were exported to SCiLS Lab software for statistical analysis, with processing using default pipelines carrying out peak picking, baseline correction and total ion current normalization, to remove systematic artifacts affecting mass spectral intensity.

Tumor and benign regions were initially compared by intensity plots. Following this, regions of cancer vs. benign tissue for each tumor sample were compared using receiver operating characteristic (ROC) curves calculated using SCiLS software to determine subsets of significant discriminatory peaks (*p* < 0.05), ranked by their area-under-the-curve (AUC) values. Only peptides with an AUC ≥ 0.7 between cancer and benign tissue were selected for further analysis. The *m/z* values of qualifying peptides were referred to the reference library of peptide IDs with corresponding *m/z* values generated by LC-MALDI-MS/MS, and were incorporated into combined lists from each tissue in the MSI experiment.

### Reference Peptide Library

LC-MALDI analysis was performed to generate a reference library of peptides present in the antigen-retrieved, trypsin-digested tissue samples, to be subsequently matched to peptides of interest found in MSI experiments. A Thermo Ultimate 3000 nano-UPLC (Thermo Fisher) was coupled to a Bruker Proteineer fcII spotting robot (Bruker Daltonics) to deposit eluent onto 384 or 1,536 sample AnchorChip MALDI target plates under conditions as previously described (14). MALDI-MS and MS/MS spectra were acquired on an UltrafleXtreme spectrometer using CHCA as matrix. Bruker flexAnalysis was used for spectral processing with protein identification performed with Proteinscape 3.0 via a MASCOT database search for human tryptic peptides as described (14).

### Survival and Network Analyses

Kaplan-Meier survival analysis was conducted using the online tool, Kaplan-Meier Plotter (15), which analyzes data from over 5,000 breast cancer patients (kmplot.com). For TNBC tumors, the selected parameters were ER-negative, PR-negative, HER2-negative, phenotype basal. For ER-positive tumors, the selected parameter was ER-positive. Only data for recurrence-free survival (RFS) were analyzed as sample numbers were too low for overall survival analyses. Cut-off values for high vs. low expression were set to auto-select. Network analysis was conducted using STRING (Search Tool for the Retrieval of Interacting Genes) v.10.5 (string-db.org) (16). When few interactions were discovered using the primary data (14 genes), EGFR was added manually to enhance the network.

### Data Accessibility

The mass spectrometry (LC-MALDI and MALDI imaging) data have been deposited to the ProteomeXchange Consortium via the PRIDE (17) partner repository with the dataset identifier PXD013397. Other data used and/or analyzed during the current study are available from the corresponding author upon reasonable request.

## Results

Two of the 10 patient samples failed to yield any identifiable peptides that could discriminate with a ROC AUC >0.7 between tissue designated as benign or cancerous by histopathological examination. Of the remaining 8 patient samples, we produced a shortlist of 14 proteins (referred to here by their gene names) that were identified in at least 3 samples each and discriminated between benign and cancerous tissue with a ROC AUC of >0.7 in each case (Table 1). COL1A2 (collagen alpha-2(I) chain) was identified by the largest number of distinct peptides (7) and was discriminatory in 7 out of 8 tissues. Based on data in Table 1 we speculate that the quality of individual tissue sections might be one factor in determining ROC AUC values, since some samples (e.g., #1, #6, and #7) gave consistently high AUC values (>0.9) for a series of peptides, whereas others (e.g. #3, #4, and #5) consistently gave lower values. An enlarged version of Table 1, including the sequences and modifications of imaged peptides, is shown as Supplementary Table 1.

**Table 1:** Peptides that distinguish cancer from benign tissue in TNBC sections.

**Protein**	**Peptide *m/z* meas**.	**ROC AUC**							
		**#1**	**#2**	**#3**	**#4**	**#5**	**#6**	**#7**	**#8**
PLEKHG2	1105.564			0.873		0.774	0.887		
SOX11	1321.635	0.956				0.769	0.956		
ATIC	1465.661	0.989		0.769		0.785	0.972	0.961	
SNCAIP	1547.037	0.983				0.769	0.987	0.980	
DHRS11	1563.694	0.989					0.976	0.973	
UBR4	1565.721	0.938				0.733	0.946	0.903	
CCDC24	1586.633	0.972				0.881	0.970	0.963	
ZSWIM8	1684.741		0.783						
	1923.749	0.864	0.791						
	2339.862				0.718				
MUC4	2057.934	0.970	0.873					0.941	
RAB5A	2106.081	0.969	0.885					0.943	
TOB2	2216.158	0.981					0.840	0.934	
COL1A1	836.459			0.817					
	868.408					0.863			
	886.474			0.840		0.847			
	1297.599				0.743		0.889		
	1691.598	0.913			0.790		0.818		
COL1A2	785.412			0.772					
	840.466			0.824	0.760	0.831			
	868.399				0.766	0.881	0.813		
	1235.682					0.760	0.948		
	1562.787		0.813			0.761		0.827	
	2027.996				0.817				
	2705.261		0.861						0.780
COL6A3	1707.836	0.901	0.824			0.809	0.951	0.955	
	1731.874						0.903	0.805	

*ROC area-under-the-curve (AUC) values for peptides that distinguish cancer from benign tissue in TNBC tissue sections #1 to #8 by MALDI MS imaging. Only values >0.7 were recorded. Full peptide sequences and posttranslational modifications are shown in Supplementary Table 1. Abbreviations: PLEKHG2, pleckstrin homology and RhoGEF domain containing G2; SOX11, SRY-box 11; ATIC, 5-aminoimidazole-4-carboxamide ribonucleotide formyltransferase/IMP cyclohydrolase; SNCAIP, synuclein alpha interacting protein; DHRS11, dehydrogenase/reductase 11; UBR4, ubiquitin protein ligase E3 component n-recognin 4; CCDC24, coiled-coil domain containing 24; ZSWIM8, zinc finger SWIM-type containing 8; MUC4, mucin 4; RAB5A, RAB5A member RAS oncogene family (Ras-related small GTPase 5A); TOB2, transducer of ERBB2, 2; COL1A1, collagen type I alpha 1 chain; COL1A2, collagen type I alpha 2 chain; COL6A3, collagen type VI alpha 3 chain*.

**Figure 1:**
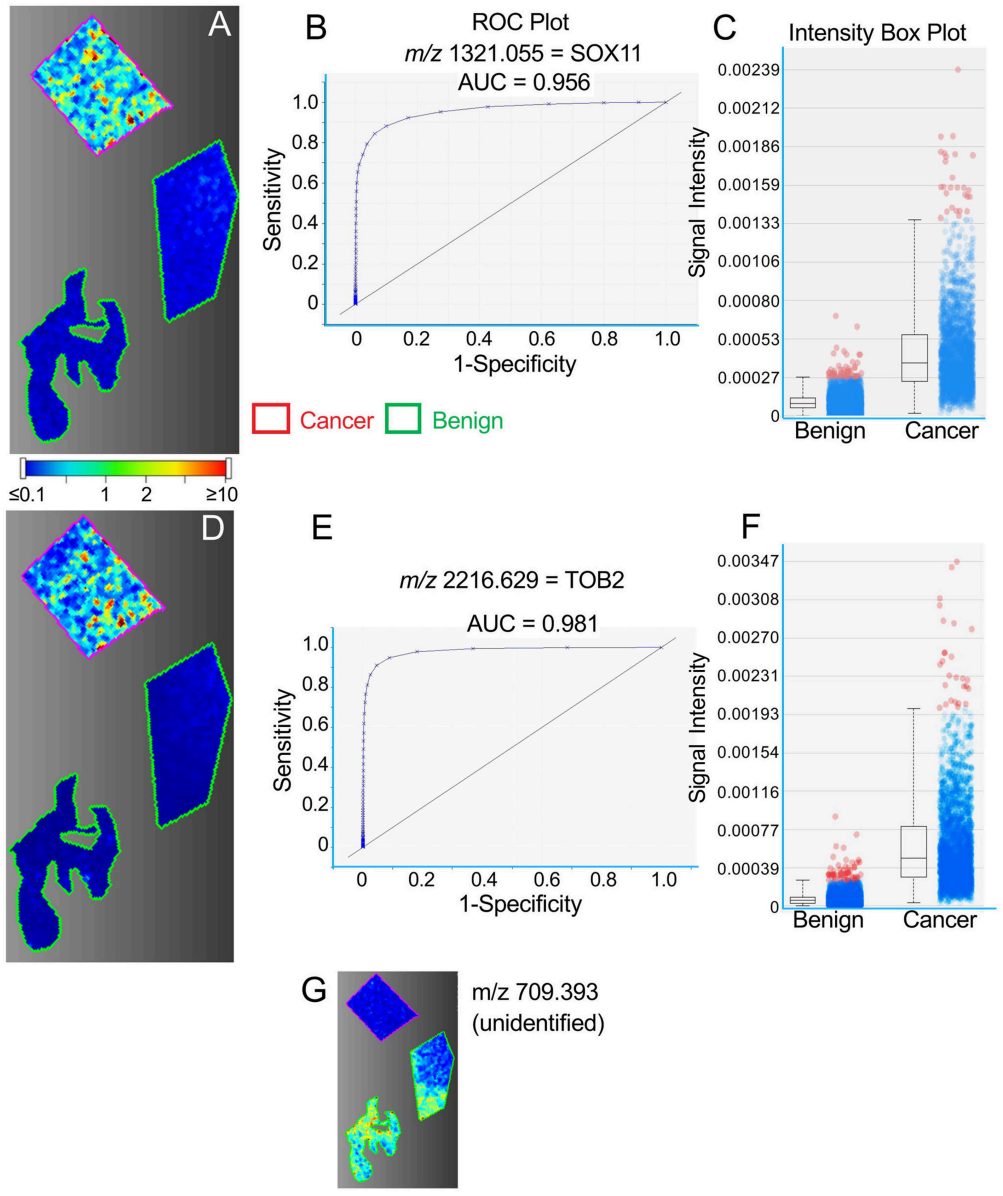
Representative MALDI images for peptides representing SOX11 **(A–C)** and TOB2 (panels **D–F**) in a single TNBC tissue section. **(A,D)** Images showing regions of cancer tissue outlined in red and benign tissue in green, within the same section. Color bar indicates relative intensity. **(B,E)** ROC curves showing the discrimination between cancer and benign tissue. **(C,F)** Intensity box plots showing relative pixel intensities for cancer and benign tissue. The boxes indicate the median (central line) and the bounds of the 2nd and 3rd quartiles; vertical broken lines indicate the range from 0 to 99th centile. Blue dots show the spread of pixel intensities, with those above the 99th centile shown in red. (**G**) Representative image of an unidentified peptide with greater abundance in benign vs. cancer tissue.

**Figure 2:**
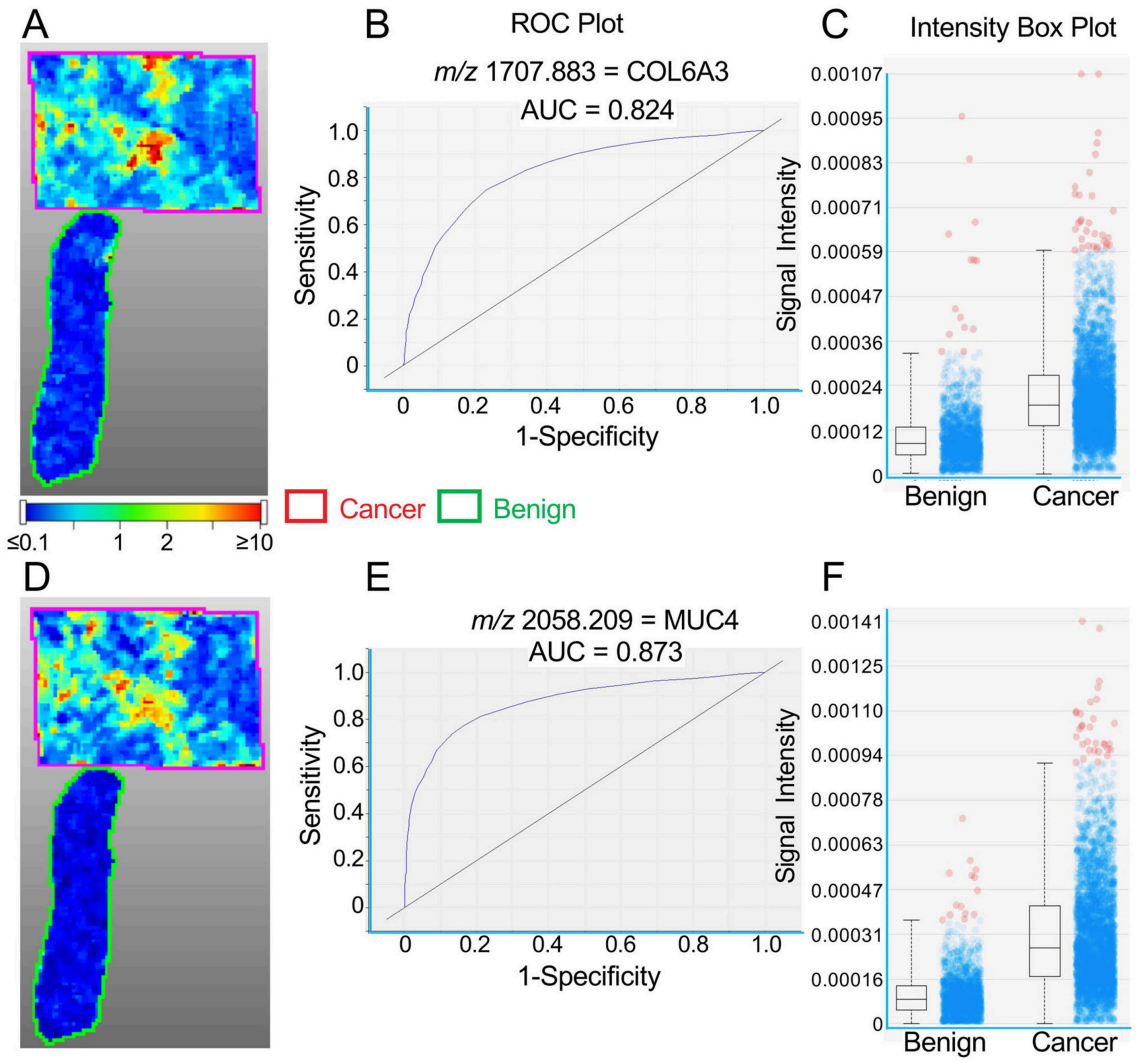
Representative MALDI images for peptides representing COL6A3 **(A–C)** and MUC4 **(D–F)** in a single TNBC tissue section. **(A,D)** Images showing cancer tissue outlined in red and benign tissue in green. Color bar indicates relative intensity. **(B,E)** ROC curves showing the discrimination between cancer and benign tissue. **(C,F)** Intensity box plots (described in Figure 1) showing relative pixel intensities for cancer and benign tissue.

**Figure 3:**
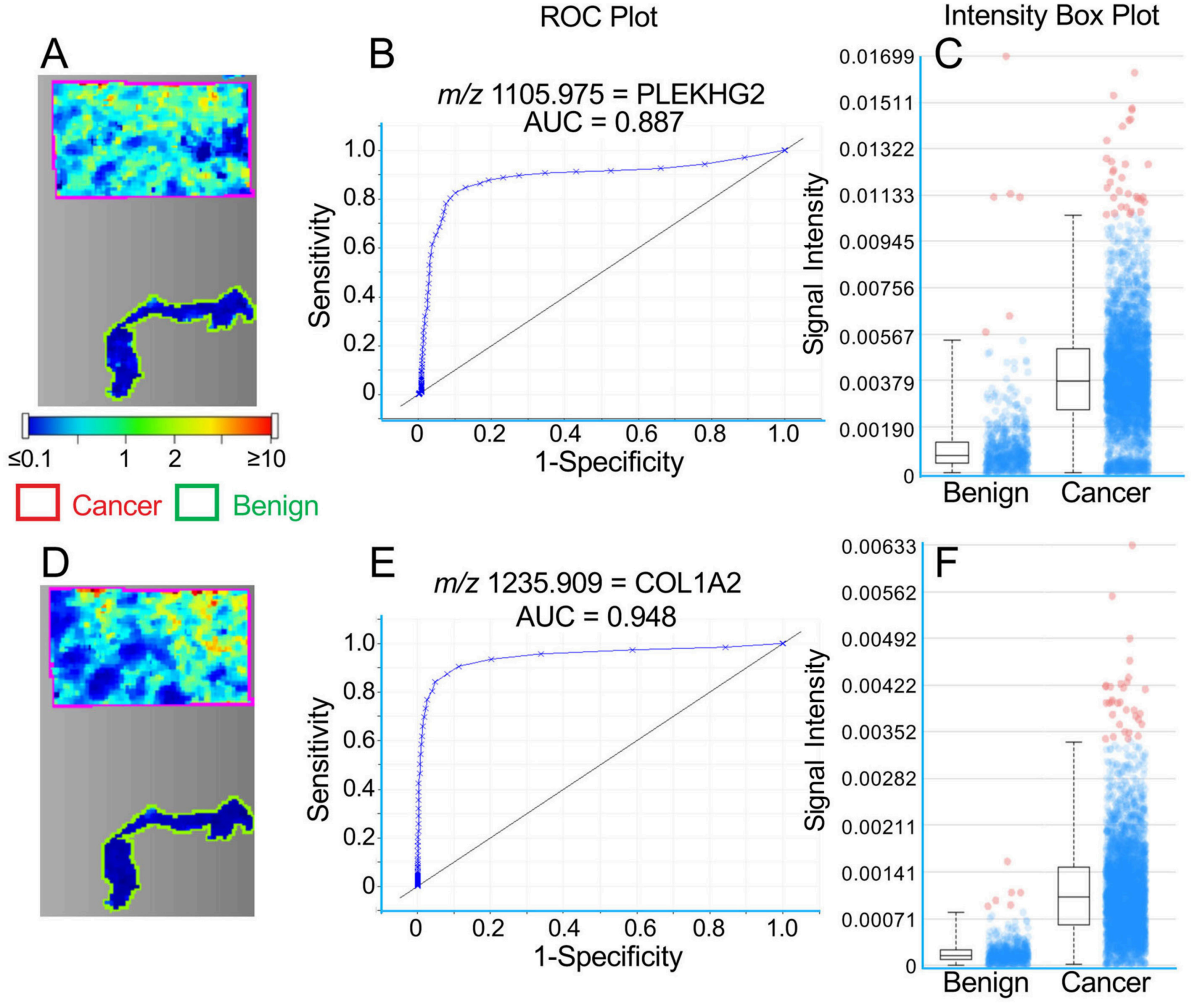
Representative MALDI images for peptides representing PLEKHG2 **(A–C)** and COL1A2 **(D–F)** in a single TNBC tissue section. **(A,D)** Images showing cancer tissue outlined in red and benign tissue in green. Color bar indicates relative intensity. **(B,E)** ROC curves showing the discrimination between cancer and benign tissue. **(C,F)** Intensity box plots (described in Figure 1) showing relative pixel intensities for cancer and benign tissue.

Figures 1–3 provide examples of discriminatory peptides imaged in tissue sections from three patient samples. For each section, regions of cancer tissue, as defined by histopathology, are outlined in red, with regions of non-cancer tissue outlined in green. In Figures 1A,D, the distribution of SOX11 and TOB2 peptides within these regions is compared in a single tissue section from patient #1, showing a high level of conformity between the two peptides (*m/z* values 1321.1 and 2216.6, respectively). Distinct “hot spots” of high intensity seen for each peptide in the cancer region are strongly concordant in this sample, in which 12 of the 14 proteins were identified. This concordance is further seen in Supplementary Figure 1 showing 10 peptides imaged in the same sample. Figures 1B,D show ROC plots for the imaged SOX11 and TOB2 peptides, indicating very strong discrimination between benign and cancerous tissue, and Figures 1C,F show box plots of pixel intensity from the images in Figures 1A,D, respectively. Contrasting with the high relative abundance of these markers in cancer tissue (and others shown in Supplementary Figure 1), Figure 1G illustrates an unidentified peptide in the same tissue (*m/z* 709.393) that is strongly downregulated in cancer vs. benign tissue.

A similar concordance between the distribution of high intensity spots is seen for COL6A3 and MUC4 peptides (*m/z* values 1707.883 and 2058.209, respectively) in Figure 2 (Patient #2), although this is less obvious for PLEKHG2 and COL1A2 peptides (*m/z* values 1105.975 and 1235.909, respectively) in Figure 3 (Patient #6).

**Figure 4:**
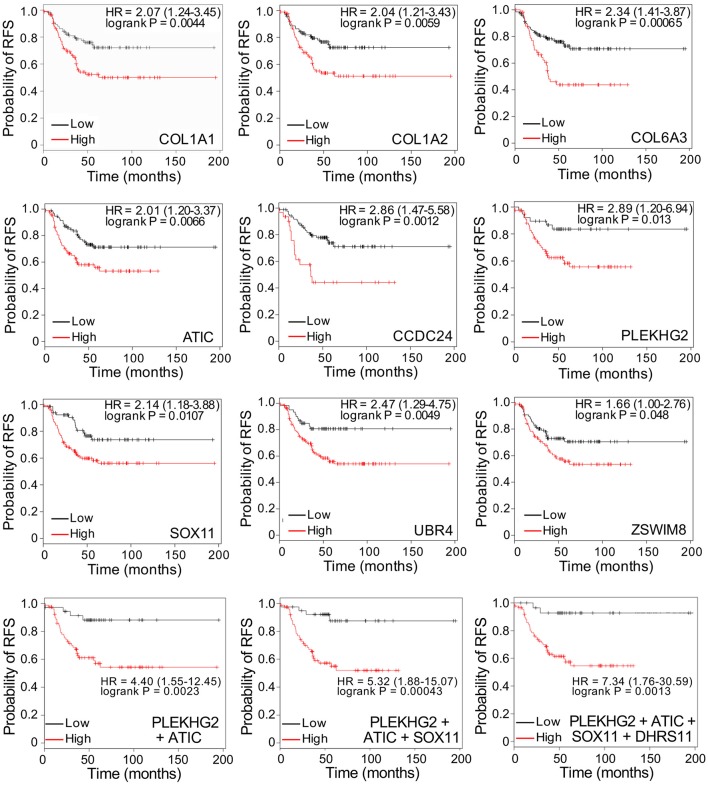
Top 9 panels: Kaplan-Meier survival curves showing significant relationships between high or low gene expression of individual putative biomarkers, discovered by MALDI MS imaging, and recurrence-free survival (RFS), in women with triple-negative, basal-like breast cancer. Bottom 3 panels: Survival curves for multigene classifiers combining 2, 3, or 4 genes as indicated. Data were generated by Kaplan-Meier Plotter (kmplot.com). HR: hazard ratio. Cut-off values defining high and low expression were auto-generated.

**Figure 5:**
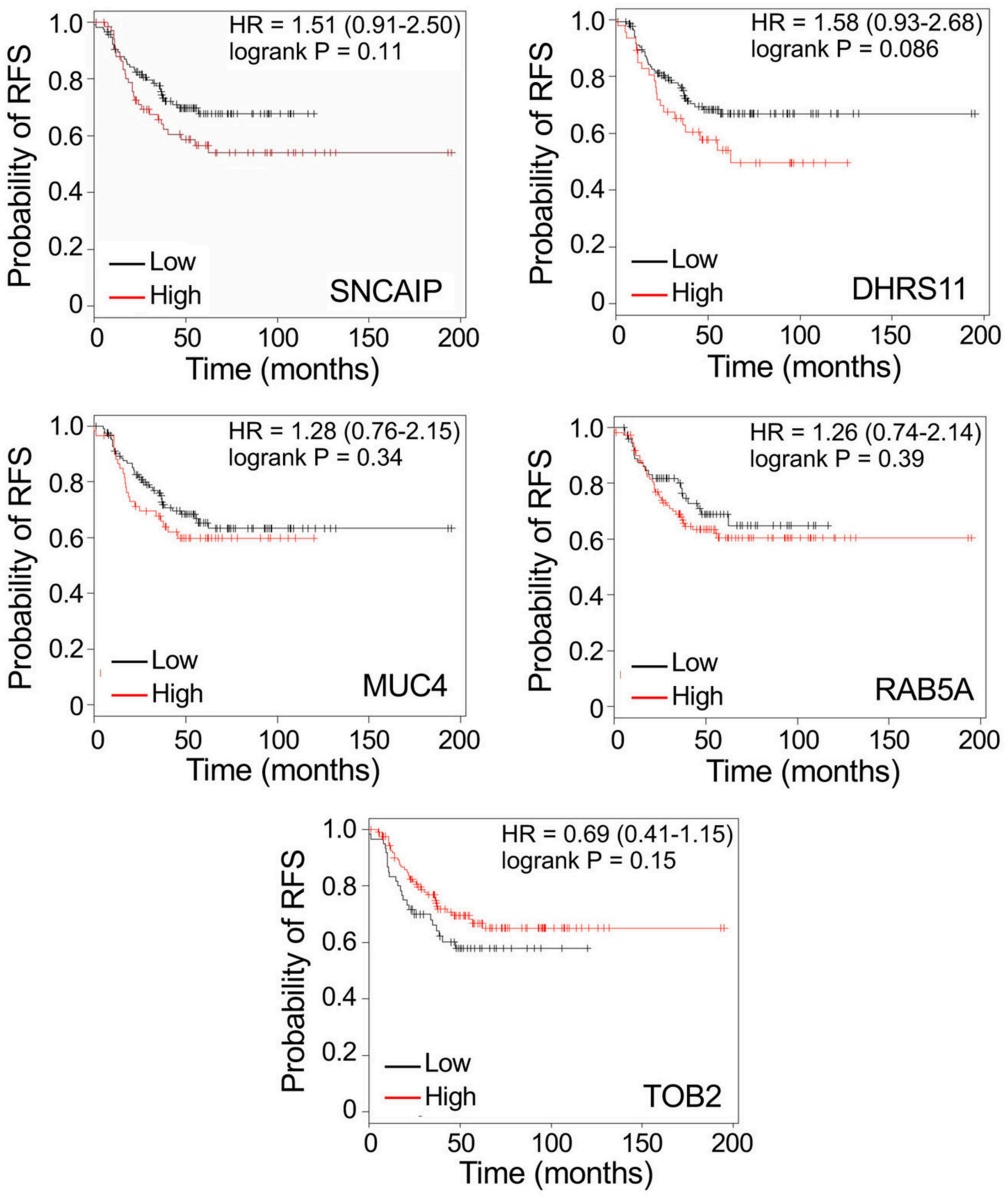
Kaplan-Meier survival curves showing non-significant relationships between gene expression of 5 putative biomarkers, discovered by MALDI MS imaging, and recurrence-free survival (RFS), in women with triple-negative, basal-like breast cancer. Data are generated by Kaplan-Meier Plotter (kmplot.com). HR: hazard ratio. Cut-off values defining high and low expression were auto-generated.

To determine whether the identification of discriminatory proteins could potentially provide markers with utility in predicting disease recurrence in patients with TNBC, we evaluated the relationship between gene expression of each of the 14 putative markers and recurrence-free survival (RFS), using public gene expression databases through the online Kaplan-Meier Plotter tool (kmplot.com). Gene expression was analyzed for tumors that were triple-negative (ER, PR, and HER2-negative), with a basal phenotype. Figure 4 (upper 9 panels) shows Kaplan-Meier survival curves related to high or low relative expression of nine genes identified by MALDI imaging of peptides derived from their protein products: *COL1A1, COL1A2, COL6A3, ATIC, CCDC24, PLEKHG2, SOX11, UBR4*, and *ZSWIM8*. In each case high expression was associated with significantly worse patient outcome (*P* < 0.05), with a hazard ratio >2 in every case except for *ZSWIM8*. Among combinations of genes tested as multigene classifiers using the same software, only combinations including *PLEKHG2* gave higher hazard ratios for poor RFS than any single gene. Figure 4 (bottom 3 panels) shows that adding *ATIC, SOX11*, and *DHRS11* to *PLEKHG2* showed increasingly strong prognostic value for poor RFS when expression of all genes was high. Notably, *DHRS11* did not show significant prognostic benefit when tested alone. Similarly, of the other 4 genes encoding proteins with differential abundance in TNBC compared to benign tissue–*SNCAIP, MUC4, RAB5A*, and *TOB2* – none showed a significant association with disease recurrence (Figure 5). Similar analyses for overall survival could not be completed owing to smaller patient numbers.

**Figure 6:**
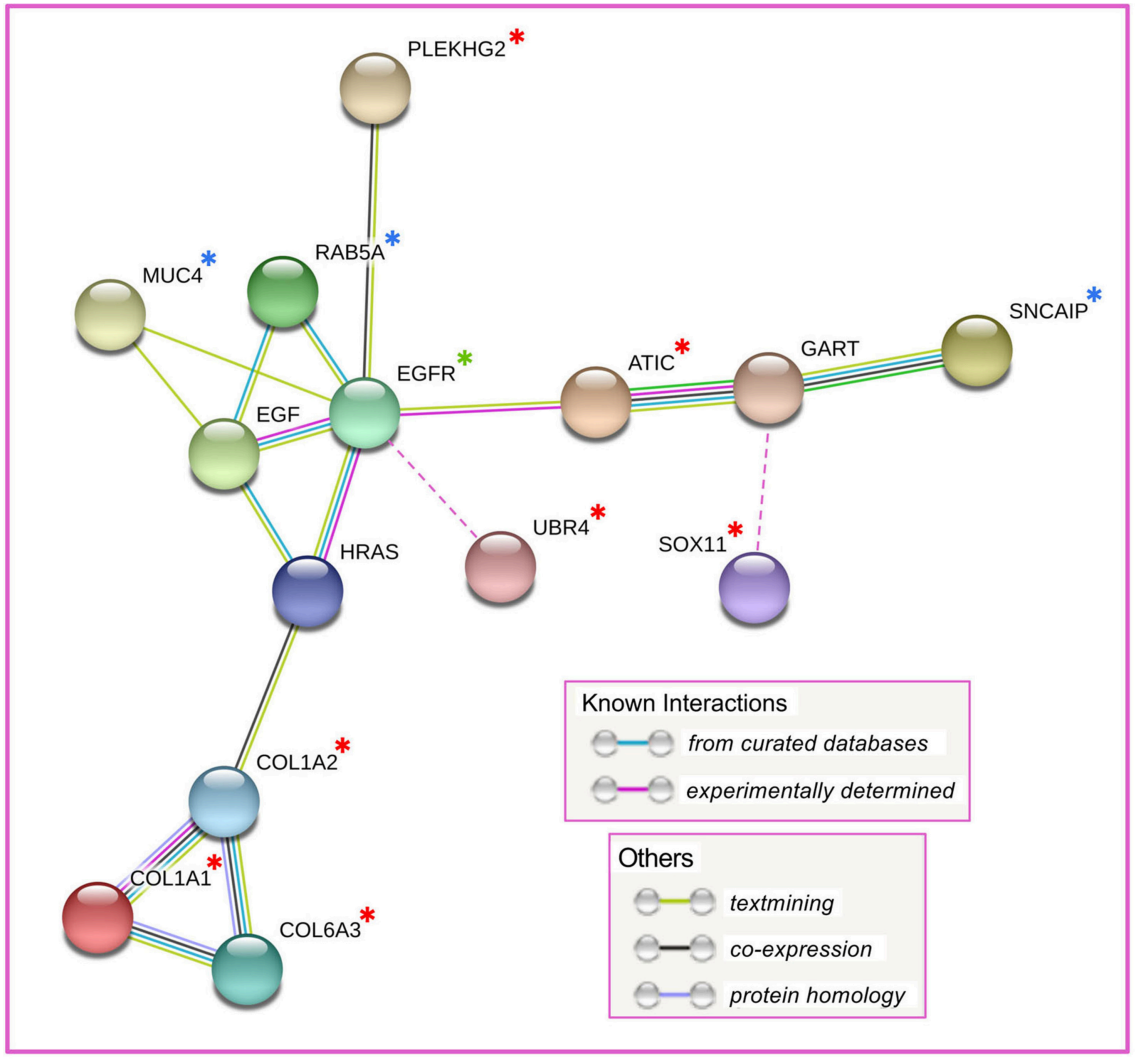
Result of STRING network analysis on 14 discovered proteins. Four proteins, not included in the network, are not shown. UBR4, not identified by STRING, was manually added to the network on the basis of reported interaction with EGFR. SOX11, not identified by STRING, was manually added to the network on the basis of reported interaction with GART. Red asterisk: proteins with putative prognostic value; blue asterisk: proteins without putative prognostic value; green asterisk: EGFR, manually added to the analysis to expand the network; unmarked proteins: added by STRING to expand the network.

Among the 14 proteins, initial network analysis using the STRING database (16) failed to reveal any known interactions, apart from relationships among the collagen subunits COL1A1, COL1A2, and COL6A3. However, when EGFR, which is a hallmark of basal-like TNBC (6), was included in the analysis (although not identified by MALDI imaging), further interactions emerged, including three proteins added by STRING–HRAS, EGF, and GART (Figure 6). The resulting network linked five of the prognostic proteins (COL1A1, COL1A2, COL6A3, ATIC, and PLEKHG2), and three of the non-prognostic proteins (MUC4, RAB5A, and SNCAIP) in pathways centered on EGFR. The prognostic protein UBR4, not included by STRING, is also associated with EGFR (18) and was manually added to the network. Similarly SOX11, reported to interact with GART (19), was manually added to the network (Figure 6). Thus, after manual curation, of the 9 discovered proteins with potential prognostic utility in TNBC, all except the two proteins without known functions (CCDC24 and ZSWIM8) could be functionally linked in a network centered on EGFR.

Although the discovery samples were all TNBC tumors, the putative biomarkers might not be specific for TNBC. Therefore, we tested the potential prognostic value of the nine markers shown in Figure 4, in ER-positive samples. Table 2 compares logrank *P*-values and hazard ratios for triple-negative, basal samples and ER-positive samples. Only two genes—SOX11 and ATIC—showed significant *P*-values for worse recurrence-free survival in patients with ER-positive tumors, and with much lower hazard ratios. Two other genes—CCDC24 and ZSWIM8—showed significance for improved recurrence-free survival in ER-positive tumors. This suggests that several of the putative biomarkers for disease recurrence in patients with triple-negative, basal breast cancer may be specific for this form of the disease.

**Table 2:** Association between high expression of nine putative biomarkers and recurrence-free survival in breast cancer patients.

		**Triple-negative, basal**		**ER-positive**	
**Gene**	**Full name, Synonyms**	**logrank P**	**HR**	**logrank P**	**HR**
*COL1A1*	Collagen type I alpha 1 chain	0.0044	2.07	0.22	0.90
*COL1A2*	Collagen type I alpha 2 chain	0.0059	2.04	0.054	0.85
*PLEKHG2*	Pleckstrin homology and RhoGEF domain containing G2	0.013	2.89	0.21	1.21
*SOX11*	SRY-box 11	0.0107	2.14	0.0059	1.27
*ATIC*	5-aminoimidazole-4-carboxamide ribonucleotide formyltransferase/IMP cyclohydrolase; *PURH, AICARFT*	0.0066	2.01	0.028	1.22
*UBR4*	Ubiquitin protein ligase E3 component n-recognin 4	0.0049	2.47	0.096	0.86
*CCDC24*	Coiled-coil domain containing 24	0.0012	2.86	0.00039	0.58
*COL6A3*	Collagen type VI alpha 3 chain	0.00065	2.34	0.18	1.12
*ZSWIM8*	Zinc finger SWIM-type containing 8; *KIAA0913*	0.048	1.66	0.0056	0.79

*Comparison of the association between high expression of nine putative biomarkers and worse recurrence-free survival in patients with either triple-negative, basal-like breast cancer or estrogen receptor-positive breast cancer. Data refer to the probability of worse recurrence-free survival for high vs. low tumor gene expression of each marker (derived from Affymetrix microarrays), with auto-selection of the best cut-off point. P-values and hazard ratios (HR) are derived from Kaplan-Meier survival analyses generated by Kaplan-Meier Plotter software (kmplot.com). Triple-negative refers to ER-, PR-, and HER2-negative*.

## Discussion

Breast tumors negative for ER, PR, and HER2 represent about 15% of all breast cancer diagnoses and have ~80% concordance with basal-like breast cancer (6). Recent classification based on histopathology and gene expression analysis (termed TNBCtype-4) defines four TNBC subtypes: basal-like 1, basal-like 2, mesenchymal, and luminal androgen receptor-like (LAR), with no significant differences in relapse-free survival among the four subtypes (20). Similarly, TNBC patient stratification according to PAM50 molecular profiling shows no differences in relapse-free survival (20). The development of new TNBC markers, beyond basal markers such as CK5/6 (3) and CK17 (2), to assist in the prediction of disease recurrence may, therefore, be of clinical benefit. In general, patients with TNBC commonly relapse within the first few years (6) and have considerably worse survival outcomes than those with other types of breast cancer (21). A recent survey of over 150,000 breast cancer patients found that those with TNBC had lower overall and disease-specific survival rates at every disease stage than seen for other types of breast cancer, after adjustment for age, race, tumor grade and treatment (5). Therefore, it would be beneficial to develop specific tissue biomarkers to aid in the management of the disease.

In recent years, MALDI-MSI of tryptic peptides generated from FFPE sections has been increasingly used for tumor classification and the discovery of putative disease biomarkers (22–25). The use of FFPE sections rather than fresh-frozen tissues extends the technique to a vast store of archival pathology specimens. We have previously demonstrated using fresh-frozen tissue lysates that the proteomic discovery of proteins with strong differential abundance between cancerous and adjacent benign tissue can be a fruitful source of potential prognostic markers (26). Compared to the analysis of lysates, the use of MALDI-MSI provides the opportunity to confine the analysis of heterogeneous tumors samples to discrete regions that have been defined histopathologically as cancer or non-cancer, in effect removing the need for laser capture microdissection. In this study we used a small discovery cohort of 10 FFPE sections, 8 of which yielded differentially abundant proteins by MALDI-MSI. The analytical process consisted of two steps: discovery by MALDI-MSI of tryptic peptides with differential abundance in multiple cancer samples, compared to their corresponding benign tissue, and identifying those with the greatest discriminatory power by reference to a library of characterized peptides, constructed by LC-MALDI-MS/MS analysis of the same tissues. Using ROC area-under-the-curve (AUC) values as a measure of discrimination, we speculate that the quality of each tissue section may have been a contributing factor in determining the range of measured ROC AUC values, with some tissues giving higher ROC AUC values for a series of markers, than others. The failure of two sections to contribute any discriminatory proteins probably relates to the heterogeneity and/or low quality of the small samples.

Using the criterion that each protein should be identified in at least 3 tissues with a ROC AUC >0.7, we developed a short-list of 14 discriminatory proteins, 10 of which were identified by a unique peptide. While there are undoubtedly further proteins that would meet these criteria, these remained unidentified owing to the absence of an identifying peptide in our reference library. This might have been overcome by increasing the number of LC-MALDI-MS/MS analytical runs to add to the library. We chose to use LC-MALDI-MS/MS rather than, for example, LC-ESI-MS/MS, to build the library on the assumption that using the same ionization technique and instrument (Bruker UltrafleXtreme) for imaging and identification runs would enhance the identification rate. This is supported by a recent study comparing different methods of peptide ionization in MS, which found only about 40% overlap in identified peptides from identical samples between MALDI and ESI, with amino acid composition and associated variables such as isoelectric point and hydrophobicity affecting the relative discovery of peptides by the two methods (27). Interestingly, for tryptic peptides, MALDI-MS favors peptides with a C-terminal arginine, whereas ESI-MS preferentially detects peptides with C-terminal lysine (27).

Among many of the 14 proteins, the strong concordance of their distribution patterns revealed by MALDI-MSI, as exemplified in Supplementary Figure 1, supports the idea of their concordant upregulation in specific clusters of cells within TNBC tumor tissue. Nevertheless, a functional network was not evident until EGFR was manually added to the STRING analysis, revealing interactions among 10 of the 14 proteins. The addition of EGFR to the putative network was based on its reported abundance in many basal-like TNBC tumors (6), rather than on our experimental findings. We did not identify EGFR peptides as discriminatory in any of our imaging analyses. Indeed, high EGFR gene expression is not prognostic for poor survival in basal-like, triple-negative breast cancers according to the survival analysis tool used in this study (Kaplan-Meier Plotter). When EGFR was added manually, two further interacting proteins, UBR4 and SOX11, were discovered by manual curation of the network. Interactions within this putative network are discussed further below.

Gene expression analysis of the identified proteins showed that 9 had potential prognostic significance in basal-like TNBC, in 8 cases with a hazard ratio >2 for poor RFS related to high expression. In contrast, when evaluated in ER-positive tumors, two were significantly associated with improved RFS and the others were non-significant or had hazard ratios close to 1 for poor survival, suggesting that the potential prognostic utility of the markers may be confined to patients with TNBC tumors. These suggestive results will need to be confirmed by independent techniques such as immunohistochemistry before these putative biomarkers could be considered for introduction into clinical practice.

### Interactions Among Discovered Proteins

Many of the putative biomarkers identified in this study have potential functional links through EGFR. Although not discovered in our MALDI MSI study, this protein was manually added to the network analysis because of its central role in basal-like TNBC (6). EGFR is overexpressed in a majority of TNBC tumors, where it was found to be prognostic for poor disease-free and overall survival in some studies (28). However, anti-EGFR agents have not proved effective as monotherapies (29). Among the proteins with functional links to EGFR, PLEKHG2 (pleckstrin homology domain-containing family G member 2) is a guanine nucleotide exchange factor for the small GTPases Rac and Cdc42. It is known to be activated by Ser/Thr phosphorylation in response to EGFR stimulation (30), although the phosphorylation site in the peptide that identified PLEKHG2 as upregulated in three TNBC tissue samples (phosphorylated at Ser1304–Supplementary Table 1) has not previously been identified. PLEKHG2 is involved in trancriptional regulation and control of cell morphology (31). Although not previously implicated in TNBC, PLEKHG2 is believed to be involved in sphingosine-1-phosphate signaling (31), which we have shown to activate EGFR and to contribute to oncogenic signaling in TNBC cell lines (32). The small GTP binding protein RAB5A, upregulated in TNBC tissues but not significant for recurrence-free survival in our analysis, is functionally linked to PLEKHG2 through a small GTPases protein interaction network (33). It is upregulated by EGFR in the TNBC cell line MDA-MB-231 and is associated with lymph node metastasis in breast cancer patients (34).

The ubiquitin E3 ligase UBR4 (ubiquitin protein ligase E3 component N-recognin 4), also known as p600, was not detected in the STRING network analysis but was added manually as it has clear links to EGFR. Described as a component of the EGFR interactome, its interaction with EGFR increases (like other E3 ubiquitin ligases) in response to EGF stimulation (18). This is thought to act in EGFR internalization and perhaps degradation (18). UBR4 is also reported to be involved in cytoskeletal organization and has a role in cell migration and survival (35), which may be related to its upregulation in TNBC.

ATIC (aminoimidazole carboxamide ribonucleotide transformylase/inosine monophosphate cyclohydrolase), required for *de novo* purine biosynthesis, is important in cell proliferation and an inhibitor of its dimerization was inhibitory to breast cancer cell growth (36). Purine biosynthesis inhibitors have previously been proposed as cancer therapeutics (37), and our data suggest ATIC as a potential target warranting further investigation. ATIC expression is co-regulated after chemotherapy treatment with another enzyme of purine biosynthesis, GART (trifunctional purine biosynthetic protein adenosine-3) (38), which is linked in the network to two other discovered proteins, SNCAIP and SOX11 (see below). Among the other discovered proteins, MUC4 (mucin 4), although not identified as prognostic in our Kaplan-Meier analysis, has been previously associated with the aggressive phenotype of TNBC, acting at least in part by upregulation of EGFR (39).

Of the other identified proteins associated with breast cancer, COL1A1 and COL1A2, the two components of type I collagen, were shown to be upregulated in invasive breast cancer, with a potential role in spinal metastasis, although not proposed as potential targets (40). In contrast, another study found that type 1 collagen fibers increased in human MDA-MB-231 TNBC xenograft tumors when the hypoxia factors HIF-1α or HIF-2α were downregulated (41), consistent with the earlier finding of reduced collagen fibers in hypoxic tumor regions (42), but apparently contradicting a role for type 1 collagen in TNBC invasion and metastasis. Since metastases from ER-/PR- breast tumors are reportedly less likely to be skeletal compared to those from receptor-positive tumors (43), the precise importance of COL1A1/COL1A2 upregulation in TNBC remains unclear. The type 1 collagen subunits are also upregulated in metastatic ovarian cancer (44), invasive bladder cancer (45), cholangiocarcinoma (46) and other malignancies, where they have been proposed to have potential prognostic significance. An alternative mRNA transcript of the other collagen-isoform gene, COL6A3, was also reported to be upregulated in almost all breast cancer samples (47) although, again, the significance of this finding for TNBC has not been explored.

The transcription factor SOX11, potentially of prognostic value but not initially identified in the STRING analysis, has previously been shown to have a role in breast cancer growth and invasion, and in regulating the basal-like phenotype (48). SOX11, described by others as a marker of poor prognosis in basal-like breast cancer (48)—although another study unstratified for subtype did not show this effect (49)—has been proposed as a therapeutic target in breast (48) and other (50) cancers. As noted above, SOX11 can be included in the STRING network through its interaction, identified by coimmunoprecipitation and LC-MS/MS (19) with the purine biosynthetic protein GART. Thus, of the 9 proteins we have identified by MALDI MSI as having potential prognostic value in TNBC, only the two without known function (CCDC24 and ZSWIM8) were unable to be included in a single interaction network.

In conclusion, we have used MSI to identify a novel network of proteins that strongly discriminate between cancer and benign tissue in TNBC, and may be suitable for evaluation as biomarkers of worse disease recurrence in patients with this breast cancer subtype. Although extensive clinical validation is required before any of these proteins could be introduced into clinical practice, gene expression analysis supports the possibility of prognostic utility for these proteins, perhaps with selectivity for TNBC compared to other forms of breast cancer. This study reiterates the power of MSI as a biomarker discovery tool and offers the possibility of enhanced prognostic capability to assist in the management of patients with TNBC, as well as some putative molecular targets against which novel therapies might be developed.

## Ethics Statement

This study has been approved by the Human Research Ethics Committee of the Northern Sydney Local Health District (NSLHD), NSW, Australia. Patient consent was not required for the analysis of deidentified archival FFPE samples.

## Author Contributions

RB conceptualized and supervised the project. LP and RB acquired and analyzed the data. LP contributed to mass spectrometry data interpretation. RB interpreted the survival and network analyses data. AG contributed to pathology data interpretation. RB wrote the manuscript. All authors read, edited, and approved the final manuscript.

### Conflict of Interest Statement

The authors declare that the research was conducted in the absence of any commercial or financial relationships that could be construed as a potential conflict of interest.

## Abbreviations

TNBC: Triple-negative breast cancer
MSI: mass spectrometry imaging
CK: cytokeratin
FFPE: formalin-fixed paraffin-embedded
ROC AUC: Receiver operating characteristic area under the curve.
