# Supplementary material for: Novel Prognostic Markers in Triple-Negative Breast Cancer Discovered by MALDI-Mass Spectrometry Imaging
Source: Front Oncol. 2019 May 14;9:379. doi: 10.3389/fonc.2019.00379 (PMC6527753; doi:10.3389/fonc.2019.00379)
Supplement: Supplementary file 2 [file Data_Sheet_1.PDF]

Supplementary Material

Novel Prognostic Markers in Triple-Negative Breast Cancer Discovered by MALDI-Mass Spectrometry Imaging

Leo Phillips, Anthony J. Gill and Robert C. Baxter*

Correspondence: Robert C Baxter, DSc: robert.baxter@sydney.edu.au

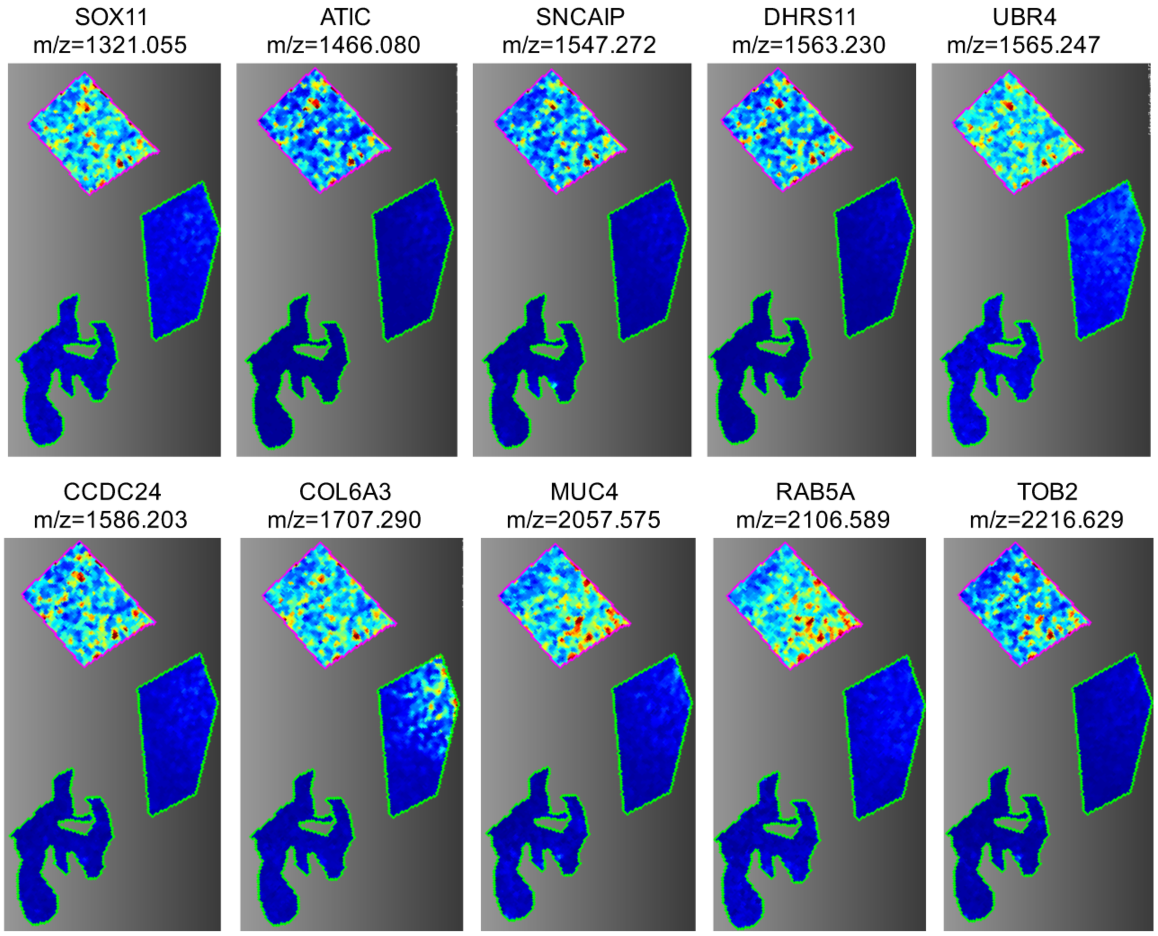

Supplementary Figure 1. Representative MALDI images of 10 peptides, as indicated, in a single TNBC tissue section, showing strong concordance in the distribution patterns for different peptides.
