# Supplementary material for: Novel Prognostic Markers in Triple-Negative Breast Cancer Discovered by MALDI-Mass Spectrometry Imaging
Source: Front Oncol. 2019 May 14;9:379. doi: 10.3389/fonc.2019.00379 (PMC6527753; doi:10.3389/fonc.2019.00379)
Supplement: Supplementary file 3 [file Data_Sheet_2.PDF]

Supplementary figure for review purposes only

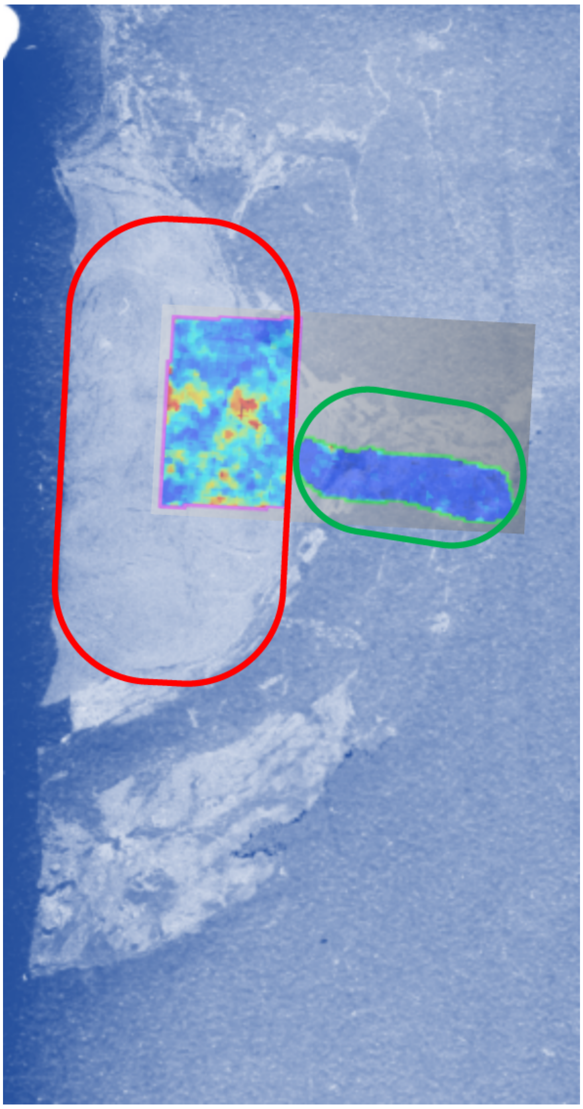

Figure shows an example of the actual imaged areas (bounded by red for cancer, green for non-cancer) within the larger regions of the tissue section designated as either cancer (red) or benign (green) by the pathologist.
